# Possible drug-drug interactions among elderly patients receiving antiviral therapy for chronic hepatitis B

**DOI:** 10.3325/cmj.2024.65.305

**Published:** 2024-08

**Authors:** Mehmet Çelik, Yusuf Arslan, Taylan Önder, Sevil Alkan, Ahmet Şahin, Fethiye Akgül

**Affiliations:** 1 Department of Infectious Diseases and Clinical Microbiology, Harran University, Sanliurfa, Turkey; 2 Department of Infectious Diseases and Clinical Microbiology, Batman Training and Research Hospital, Batman, Turkey; 3 Department of Infectious Diseases and Clinical Microbiology, Osmaniye Kadirli State Hospital, Osmaniye, Turkey; 4 Department of Infectious Diseases and Clinical Microbiology, Canakkale Onsekiz Mart University, Canakkale, Turkey; 5 Department of Infectious Diseases and Clinical Microbiology, Dr. Ersin Arslan Training and Research Hospital, Gaziantep, Turkey

## Abstract

**Aim:**

To identify possible drug-drug interactions in patients taking medications for other comorbidities while on antiviral therapy for chronic hepatitis B.

**Methods:**

The study enrolled patients with chronic hepatitis B aged ≥60 years who were treated with antiviral therapy in five hospitals in Turkey between January 1 and March 1, 2023. The Lexicomp® Drug Interactions program was used to identify possible drug-drug interactions.

**Results:**

The study included 213 patients (119 [55.9%] men). The mean age was 68.5 years. A potential drug-drug interaction was identified in 112 patients (52.6%). The most common type of interaction was type C (“follow the treatment”) (71.54%). The number of potential drug-drug interactions increased with an increase in the number of drugs used by the patients. A robust and affirmative correlation was observed between the number of medications used and the number of possible drug-drug interactions (r = 0.791, *P* < 0.001). Adverse interactions (interactions of types C and D, 3.7% of cases) were limited to patients receiving tenofovir disoproxil fumarate.

**Conclusion:**

Nonsteroidal anti-inflammatory medications should be used cautiously in elderly patients with chronic hepatitis B treated with tenofovir disoproxil fumarate due to the increased risk of renal toxicity.

Chronic hepatitis B (CHB) infection is one of the main global causes of death. The World Health Organization (WHO) estimates that 820 000 people worldwide die from cirrhosis and hepatocellular carcinoma each year, while 296 million are infected with hepatitis B virus (HBV) (1).

Antivirals used to treat HBV infection effectively suppress the virus and improve survival and the quality of life (2). Unfortunately, no treatment strategy offers total virological recovery (2,3). Antiviral drugs lower the risk of hepatocellular carcinoma, liver-related death, and the development of cirrhosis (4,5). Currently, two interferon-based drugs used subcutaneously and seven nucleos(t)ide analog drugs used orally are approved for the treatment of CHB. The first-line drugs for the treatment of HBV infection are tenofovir disoproxil fumarate, tenofovir alafenamide, entecavir, and pegylated interferon, while the use of adefovir, lamivudine, and telbivudine is decreasing due to drug resistance. Interferon-based therapies are considered first-line choices, but their use is restricted because of their substantial adverse effect profile, poor tolerability, and subcutaneous delivery (6). Both tenofovir disoproxil fumarate and entecavir have a good safety profile (2,7,8).

Multiple chronic diseases complicate treatment management and harm health outcomes (9). Polypharmacy is frequent among the older population with multimorbidity. It may result in adverse medication responses, extended hospital stays, rehospitalization, or death. Drug-drug interactions (DDIs) and drug-disease interactions raise the risk associated with drug use. Elderly individuals are more vulnerable to drug interactions due to reduced hepatic and renal functions, low lean body mass, and limited movement (9-12).

DDIs are interactions between medication combinations that may produce unfavorable outcomes yet are intended to have additive or synergistic effects for various purposes. DDI is theoretical and has no practical implications. The term “potential drug-drug interaction” (pDDI) refers to the co-administration of two or more medications that interact and may have therapeutic implications (13). Healthcare providers should prevent harmful DDIs by avoiding combinations that could negatively interact, monitoring for early diagnosis, using computerized imaging and decision support systems, and educating patients about prescription and over-the-counter medications. Certain databases assist in defining pDDIs, often using severity indicators such as none, minor, severe, or contraindication, enabling physicians to take appropriate action (14). Among five distinct DDI algorithms, Micromedex® and Lexi-Interact® (Lexicomp®Drug Interactions) performed best in terms of pDDI determination (15). Although there are numerous studies on DDIs, studies on DDIs between antiviral agents and other drugs used by older patients receiving treatment for hepatitis B are limited. Therefore, we sought to identify the pDDIs of patients with CHB infection treated with antiviral therapy and taking medications due to other comorbidities.

## Patients and methods

### Study design

This multicenter retrospective study enrolled patients ≤60 years old treated with antiviral therapy for CHB in Clinical Microbiology and Infectious Diseases clinics of five hospitals in Turkey between January 1, 2023, and March 1, 2023. Age, sex, chronic diseases, antiviral treatment for CHB, and other medication data were recorded.

Inclusion criteria were age ≤60 years, antiviral treatment for CHB, and taking at least one medication for chronic diseases. The exclusion criterion was the use of topical, ophthalmic, and intranasal medications.

### Evaluation of pDDI

Data on all drugs taken by the patients were obtained from hospital records. The pDDIs of all the drugs between themselves and with CHB antiviral treatments were determined by using the Lexicomp® Drug Interactions program. This program contains general information about pDDIs, interaction mechanisms, and patient management, and interactions are classified as type A, B, C, D, and X (Table 1). It was chosen for its comprehensive data on drug interactions, user-friendly interface, and reliability in identifying clinically significant drug interactions. The program's extensive database covers various medications relevant to the study population (16). The study complied with the Declaration of Helsinki and was approved by the Clinical Research Ethics Committee of Batman Training and Research Hospital.

**Table 1:** Risk grading of potential drug-drug interactions by Lexicomp® Drug Interactions program

Risk grading	Approach to interaction	Additional information
**A**	No known interactions	No pharmacodynamic or pharmacokinetic interactions have been demonstrated between the specified agents.
**B**	No action required	It has been demonstrated that the indicated agents may interact with each other, but there is little or no evidence of clinical concern arising from the concomitant use of these agents.
**C**	Follow the treatment	These agents may interact with each other. The benefits of the combined use of these two agents outweigh the risks. An appropriate monitoring plan should be in place to identify possible adverse effects. Dose adjustment of the drugs may be necessary.
**D**	Consider change of treatment	Clinically significant interactions between the two medicinal products have been demonstrated. Special precautions should be taken to maximize the benefits and/or minimize the risks arising from the concomitant use of the drugs. These precautions may include intensive monitoring, empirical dose changes or preference for alternative medicines.
**X**	Avoid combinations	The data suggests that these agents may interact with each other in a clinically significant manner. The risks associated with the concomitant use of these agents generally outweigh the benefits. Concomitant use of these agents should generally be avoided.

### Statistical analysis

Categorical data are presented as frequencies or ratios. The normality of data distribution was tested with a Kolmogorov-Smirnov test. Continuous data are presented as means and standard deviations or medians and ranges. The Mann-Whitney U and Kruskal-Wallis tests were used to assess the differences between continuous variables. The Fisher's test was used to assess differences between categorical variables. Correlation analysis was performed with a Spearman correlation test. A *P* value <0.05 was deemed significant. Statistical analysis was conducted with SPSS 27.0 for Windows (IBM Corp. Armonk, NY, USA).

## Results

The study enrolled 213 patients (119 [55.9%] male). The mean age was 68.5 ± 6.2 years (median = 68): 67.9 ± 5.6 years (median = 68) for men and 69.2 ± 6.8 years (median = 68) for women. The most common comorbid chronic diseases were cardiovascular diseases (n = 129, 60.5%) and diabetes mellitus (n = 87, 40.8%). The mean number of comorbid chronic diseases was 3.0 ± 1.2 (median 3).

Patients used a mean of 4.3 ± 2.8 drugs for all chronic diseases. Table 2 lists all medications and drug groups used by patients. Tenofovir disoproxil fumarate was the most prevalent antiviral drug (50.7%), followed by entecavir (36.6%), tenofovir alafenamide (10.8%), lamivudine (1.4%), and telbivudine (0.5%) (Table 3). Overall, 0.20% of interactions were type-A interactions, 12.80% were type-B, 71.54% type-C, 13.01% type-D, and 2.45% type-X (Table 4). The overall pDDI rate was 2.32 ± 4.5, with a pDDI detected in 52.6% of patients. The mean number of drugs used was 2.61 ± 0.9. In all patients who used more than six drugs, a pDDI was identified. Adverse interactions were related only to tenofovir disoproxil fumarate (3.7%). Drugs showing a pDDI with tenofovir disoproxil fumarate were ranolazine, verapamil/trandolapril, carvedilol (type C), diclofenac, and indomethacin (type D).

**Table 2:** All medications and drug groups used by patients in our study

Drug group	Drugs used
**Anti-hypertensives and cardiovascular system drugs**	telmisartan + hydrochlorothiazide, irbesartan + hydrochlorothiazide, ranolazine, trimetazidine dihydrochloride, nebivolol, metoprolol, lercanidipine, valsartan + hydrochlorothiazide, amlodipine + valsartan, olmesartan + hydrochlorothiazide, losartan + hydrochlorothiazide, ramipril, perindopril, benidipine, spironolactone, propranolol, diltiazem, torsemide, digoxin, carvedilol, furosemide, isosorbide mononitrate, indapamide, nifedipine, verapamil + trandolapril, bisoprolol
**Anti-diabetics**	metformin, empagliflozin, insulin aspart + insulin aspart protamine, insulin aspart, insulin detemir, linagliptin, gliclazide, pioglitazone, insulin glargine, glimepiride, vildagliptin, acarbose
**Anti-inflammatories/anti-analgesics**	acemethazine, etodolac, paracetamol, tramadol, fentanyl, naproxen, indomethacin
**Proton pump inhibitors/gastrointestinal regulators**	pantoprazole, domperidone, ondansetron, esomeprazole
**Anti-coagulants/anti-aggregants**	acetylsalicylic acid, clopidogrel, rivaroxaban, warfarin, apixaban
**Lipid regulators**	atorvastatin, fenofibrate, rosuvastatin
**Anti-depressants/anti-psychotics**	sertraline, quetiapine, duloxetine, escitalopram, trazodone, olanzapine, risperidone, venlafaxine
**Bronchodilators**	salbutamol, salbutamol + ipratropium bromide, salmeterol xinafoate + fluticasone propionate, formoterol fumarate + budezonide, montelukast, theophylline
**Hormones**	levothyroxine, megestrol, bicalutamide
**Anti-rheumatic drugs**	sulfasalazine, hydroxychloroquine sulfate, leflunomide
**Corticosteroids/immunosuppressives/ monoclonal antibodies**	methylprednisolone, methotrexate, mycophenolate mofetil, everolimus, tacrolimus, denosumab, rituximab, azathioprine, gemcitabine, docetaxel, dexamethasone, mitomycin-c
**Anti-epileptics**	gabapentin, sodium valproate, levetiracetam
**Urinary system and benign prostatic hypertrophy medications**	darifenacin, dutasteride, alfuzosin, tamsulosin, silodosin, doxazosin
**Other**	allopurinol, piracetam, memantine, alendronate sodium + cholecalciferol, calcium carbonate + cholecalciferol, alpha-lipoic acid, ursodeoxycholic acid, cilostazol, colchicine, pyridostigmine, rivastigmine, filgrastim, lenograstim, dapsone, betahistine, rasajilin, pramipexolexol, benserazide, donepezil, methimazole

**Table 3:** Antiviral medications taken by the participants

Drug type	N (%)
Tenofovir disoproxil fumarate	108 (50.7)
Entecavir	78 (36.6)
Tenofovir alafenamide fumarate	23 (10.8)
Lamivudine	3 (1.4)
Telbivudine	1 (0.5)

**Table 4:** Drug-drug interaction types

Interaction type	Average ± standard deviation	Percent
**A**	0.01 ± 0.09	0.20
**B**	0.30 ± 1.0	12.80
**C**	1.65 ± 3.1	71.54
**D**	0.30 ± 1.1	13.01
**X**	0.06 ± 0.3	2.45
**Total**	**2.32 ± 4.5**	

Table 5 presents correlations between the number of medications, age, sex, number of chronic diseases, and pDDI. The number of medications was categorized into three groups: ≤4 drugs, 5-7 drugs, and ≥8 drugs. The average age showed no significant differences between the groups. There were 39.9% of women in the group taking ≤4 drugs, 52.2% in the group taking 5-7 drugs, and 54.2% in the group taking ≥8 drugs. The number of chronic diseases increased significantly with the number of drugs used, and pDDI increased with the number of drugs used, especially in patients using ≥8 drugs.

**Table 5:** Correlations between the number of medications, age, sex, number of chronic diseases, and potential drug-drug interactions (pDDI)

	Number of medications taken by patients			
Age, median min-max (years)	≤4	5-7	≥8	*p*
	68 ± 6.2 68	68.0 ± 6.2 67.5	68.3 ± 6.8 69	0.991^‡^
**Sex, n (%)**				
women	57 (39.9)	24 (52.2)	13 (54.2)	0.197**^§^**
men	86 (60.1)	22 (47.8)	11 (45.8)	
**Number of chronic diseases,** average ± SD; median	2.49 ± 0.71 2*^†^	3.72 ± 0.93 4^†^	4.79 ± 1.52 4	<0.001^‡^
**Total interactions,** average ± SD; median	0.49 ± 0.8 1*^†^	3.54 ± 2.5 3.5^†^	10.87 ± 8.5 8	<0.001^‡^

*Difference compared with patients taking 5-7 medications, *P* < 0.05.

†Difference compared with patients taking ≥8 medications, *P* < 0.05.

^‡^Kruskal-Wallis (Mann-Whitney u test).

**§**χ^2^ test (Fisher’s test).

**Figure 1:**
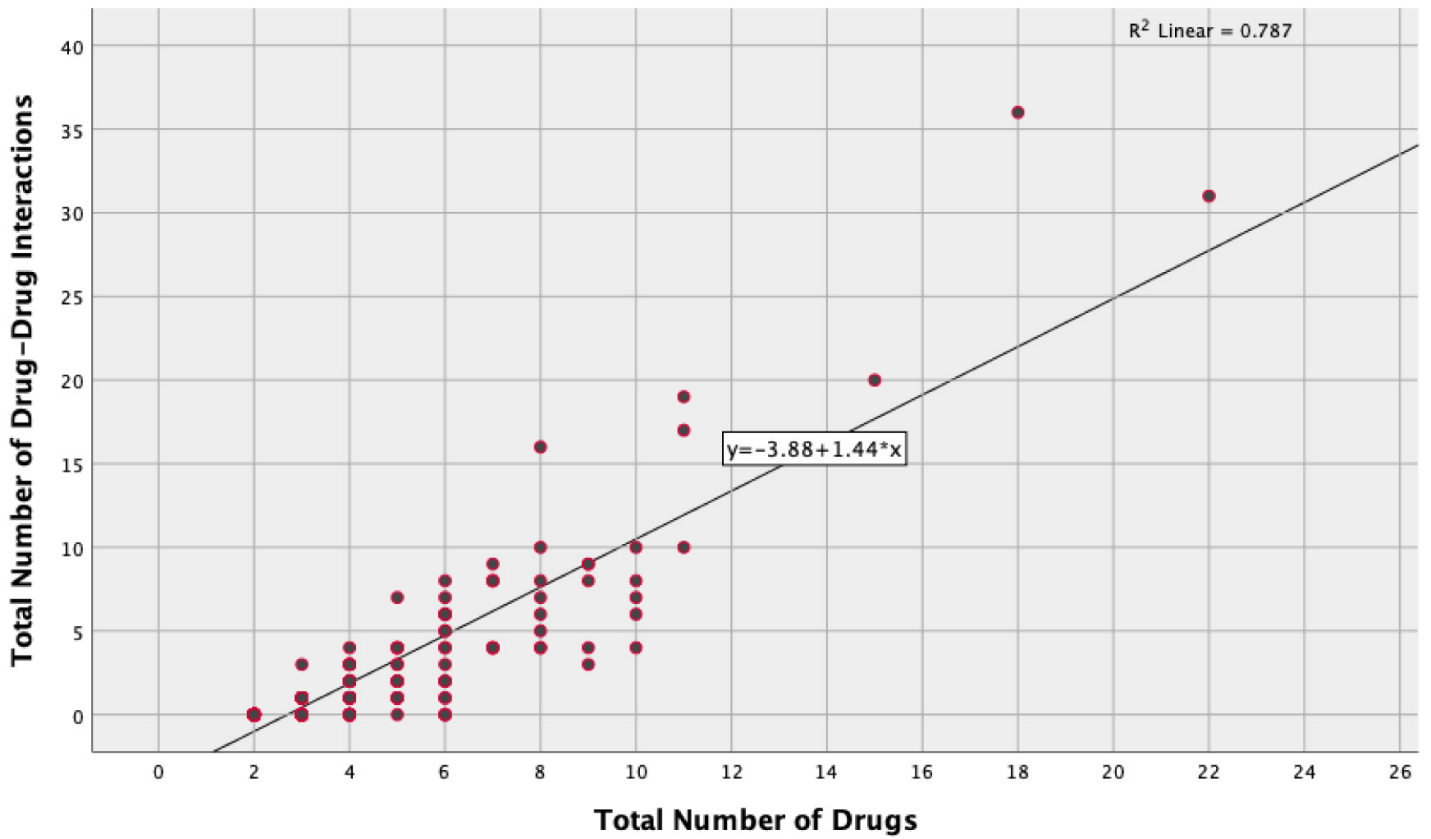
Scatter/dot plot of total possible drug-drug interactions according to the number of drugs used.

**Figure 2:**
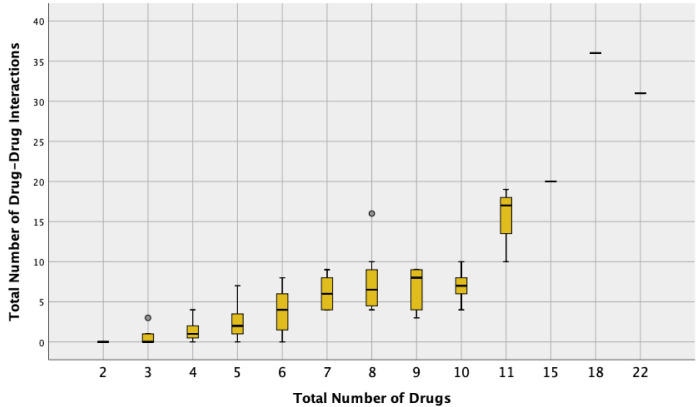
Boxplot of total drug-drug interactions according to the number of drugs used.

Medication use positively correlated with pDDIs (r = 0.846, *P* < 0.001), while chronic diseases moderately positively correlated with pDDIs (r = 0.682, *P* < 0.001) (Table 6, Figure 1-2). pDDIs were identified in patients using tenofovir disoproxil fumarate, with 4.7% of 108 patients having pDDI. Three patients had type-C pDDI, while one had type-D pDDI. Patients using all other antivirals had type-A pDDIs.

**Table 6:** Correlations between number of interactions, number of drugs, number of chronic diseases, and age

	*Spearman’s rho	Number of interactions	Number of drugs	Number of chronic diseases	Age
**Number of interactions**	r p	1			
**Number of drugs**	r p	0.846 <0.001			
**Number of chronic diseases**	r p	0.682 <0.001	0.805 <0.001		
**Age**	r p	-0.044 0.522	-0.062 0.369	-0.024 0.729	1

## Discussion

In this study, patients used an average of 4.3 ± 2.8 drugs for comorbid diseases, and for 52.6% a pDDI was identified between the antiviral agents used for hepatitis B treatment and other drugs. Type C (71.54%) was the most detected pDDI and, among the antiviral agents used for hepatitis B treatment, adverse interactions were only associated with tenofovir disoproxil fumarate (3.7%).

Adverse drug events (ADEs) can happen to anyone at any age, but they are more common in the elderly because of multimorbidity, polypharmacy, and pharmacokinetics and pharmacodynamics that result from aging and increase susceptibility to drug toxicity (17,18). Increased rates of polypharmacy have been associated with pDDIs in both outpatients and inpatients (17). In a study by Savran et al (19), the rate of pDDIs was 83.78%, the mean number of pDDIs per patient was 6.06, and polypharmacy was significantly related to pDDIs. Mousavi et al (20) observed the pDDI rate of 86.2%, with 7.6 ± 8.8 interactions per patient, and a significant relationship between the use of seven or more drugs and pDDIs. Akshaya Srikanth et al (21) showed 4.13 interactions per patient and a significant association between pDDIs and the use of three or more drugs. In our study, patients used an average of 4.3 ± 2.8 drugs for comorbid diseases. While a pDDI was found in 52.6% of patients, the number of pDDIs per patient was 2.32 ± 4.5. Also, the number of pDDIs increased as the number of drugs used increased; this increase was more pronounced in patients using eight or more drugs, and the number of medications was significantly positively correlated with the number of pDDIs.

In our study, type C (71.54%) was the most common type of interaction, while type-D interaction was observed in 13.01% and type-X interaction in 2.45%. Mousavi et al (20) most frequently observed type-C (78.6%) interactions in hospitalized patients, while a type-X interaction was found in 9.2% of participants. Dirin et al (22) predominantly observed type-C interactions (66%), and a relatively small proportion (0.14%) of type-X interactions.

In our sample, the most commonly used antivirals for CHB were tenofovir disoproxil fumarate and entecavir. Adverse interactions were related only to tenofovir disoproxil fumarate (3.7%). Tenofovir disoproxil fumarate has shown good safety and has been linked to a low risk of renal failure and decreased bone mineral density (23,24). Tenofovir is neither a substrate nor an inhibitor of cytochrome P450 (CYP) enzymes; therefore, it has a low potential for clinically relevant DDIs with drugs that are substrates or inducers/inhibitors of these enzymes (25). Similarly, entecavir, a guanosine nucleoside analog, has no effect on the CYP enzyme system and is not likely to interact with substances that do (26).

Drugs showing pDDIs with tenofovir disoproxil fumarate were ranolazine, verapamil/trandolapril, carvedilol (type C), diclofenac, and indomethacin (type D). Ranolazine, used in the treatment of stable angina pectoris, is primarily cleared by CYP3A4 (70%-85%) and is a substrate of P-glycoprotein (27). P-glycoproteins are transmembrane ATP-dependent efflux proteins. The activity of P-glycoprotein can be inhibited, induced, or altered due to polymorphism. This may lead to changes in the bioavailability and pharmacokinetic properties of drugs (28). Verapamil is widely used for treatment of hypertension, cardiac arrhythmias, migraines, and cluster headaches. It is also a potent inhibitor of P-glycoprotein function (29,30). Carvedilol is a non-selective β-blocker agent used in the treatment of mild or moderate hypertension. Carvedilol inhibits P-glycoprotein-mediated transport in the intestine (31). These P-glycoprotein-inhibiting substances may raise the blood levels of tenofovir disoproxil fumarate. Patients taking tenofovir disoproxil fumarate in combination with P-glycoprotein inhibitors should have their side effects carefully monitored (16). Tenofovir disoproxil fumarate is a prodrug that breaks down quickly in plasma to produce tenofovir. By means of proximal tubular secretion and glomerular filtration, tenofovir is eliminated unaltered. After it enters proximal renal tubular cells via human organic anion transporter 1, tenofovir is secreted into urine by multidrug-resistance proteins, in particular multidrug-resistance protein 4 (MRP4). Drug transport by MRP4 is inhibited by nonsteroidal anti-inflammatory drugs (NSAIDs). When used concomitantly with tenofovir, NSAIDs may accumulate in proximal tubular cells because of a DDI, leading to an increased risk of tenofovir disoproxil fumarate toxicity. Therefore, patients who use NSAIDs such as diclofenac and indomethacin in combination with tenofovir disoproxil fumarate should be monitored for signs of nephrotoxicity (32).

The study limitations include retrospective design, lack of access to real-time pDDI and adverse outcomes, and insufficient focus on clinical outcomes. The study enrolled only elderly chronic hepatitis B patients, which limits the generalizability of the results. Furthermore, the use of non-prescribed medications was not considered, and only one database was used for pDDI determination. Also, patients with hematologic or solid organ malignancies were underrepresented. These issues should be addressed in future prospective observational studies comparing actual DDIs with pDDIs.

To summarize, while antivirals used in CHB infection are generally safe, it is important to identify their potential interactions with drugs for other comorbidities. In this study, type-C and type-D pDDIs were identified only when tenofovir disoproxil fumarate was used, while no significant drug interactions were found with other antiviral agents. Particular attention should be paid to the use of NSAIDs with tenofovir disoproxil fumarate due to the risk of renal toxicity. Identification of PDDIs with computer databases can help clinicians to prevent possible side effects.
